# Reclassification of Low or Intermediate Cardiovascular Risk by Determining Lipoprotein(a) Levels

**DOI:** 10.3390/biomedicines13112648

**Published:** 2025-10-29

**Authors:** Alberto Cordero, José Ma Salinas, María Amparo Quintanilla, José Ma López-Ayala, Álvaro Blasco, Emilio Flores

**Affiliations:** 1 Cardiology Department, Hospital Universitario de San Juan, 03550 Alicante, Spain; 2 Grupo de Investigación Cardiovascular (GRINCAVA), Universidad Miguel Hernández, 03202 Elche, Spain; 3 Centro de Investigación Biomédica en Red de Enfermedades Cardiovasculares (CIBERCV), 28029 Madrid, Spain; 4 Servicio de Informática, Hospital Universitario de San Juan, 03550 Alicante, Spain; 5 Clinical Laboratory, Hospital Universitario de San Juan, 03550 Alicante, Spain; 6 Diagnostic Innovation in Laboratory Medicine Group, FISABIO, 46020 València, Spain

**Keywords:** risk, Lipoprotein(a), reclassification

## Abstract

**Background:** Lipoprotein(a) [Lp(a)] is a modifier of cardiovascular risk, and it should be determined at least once in a lifetime. **Methods:** Subjects with low or moderate cardiovascular risk, estimated by SCORE2, were invited to have a determination of Lp(a), and those with Lp(a) > 50 mg/dL were classified into a higher-risk category. Eligibility of statins was assessed according to treatment targets. **Results:** We analyzed 140 subjects, with a mean age of 54.3 (8.1) years and 62.9% women. The median Lp(a) was 15.2 (interquartile range: 6.7–44.5) mg/dL, and 22.1% of the cohort had Lp(a) > 50 mg/dL. No differences were observed in mean age, sex, or lipid profile in subjects with Lp(a) below or above 50 mg/dL; alkaline phosphatase (ALP) was significantly higher in subjects with Lp(a) > 50 mg/dL. After incorporating Lp(a) values into the SCORE-2 assessment, 22.6% of individuals initially of low risk were reclassified as moderate risk, and 77.4% were reclassified from moderate to high risk; moreover, 61.4% (86 subjects) were considered eligible for treatment with statins. **Conclusions:** Our results highlight that 22.1% of the subjects classified as low or moderate cardiovascular risk by SCORE-2 are reclassified to higher risk, and 61.4% were eligible for statin treatment as a result of Lp(a) testing.

## 1. Introduction

Individualized cardiovascular risk stratification is recommended for analyzing the risk of developing major cardiovascular complications and for guiding the treatment of different cardiovascular risk factors [1]. The European Society of Cardiology recommends assessing SCORE-2 in people without cardiovascular disease. This provides an estimation of individuals’ 10-year risk of developing or dying from cardiovascular disease, and subjects are classified as having low (<5% at 10 years), intermediate (5–10%), or high cardiovascular risk (>10%) [1]. Age, gender, systolic blood pressure, smoking, and non-HDL cholesterol are used for the assessment of the SCORE-2 although some situations reclassify the risk obtained by the SCORE2 scale, such as the detection of atherosclerotic plaques in the carotid arteries, microalbuminuria, or glomerular filtration rates of <30 mL/min/1.72 m^2^, and these subjects are categorized as high risk [1].

Lipoprotein(a) (Lp(a)) is a low-density lipoprotein (LDL) particle that contains an additional protein, apoLipoprotein(a) [2]. Values of Lp(a) > 50 mg/dL are associated with an increased risk of cardiovascular complications, while values > 180 mg/dL confer a risk similar to that of familial hypercholesterolemia [3,4,5]. It is estimated that approximately 6–8% of people without cardiovascular disease have values > 50 mg/dL; however, this percentage can be as high as 20% among patients with premature cardiovascular disease [6,7,8]. Lp(a) levels are genetically determined in more than 80% of cases and do not change with age or lifestyle; therefore, a single measurement in a person’s lifetime is considered sufficient to determine the cardiovascular risk associated with Lp(a) [2,3,9]. Nonetheless, several reports have highlighted the low rate of Lp(a) measurement in the population [6,7]. The 2025 update on the clinical guidelines for the management of dyslipidemias endorsed Lp(a) as an established modifier of cardiovascular risk [10].

The objective of our study was to implement Lp(a) measurements for people with low or moderate cardiovascular risk in our healthcare setting in order to assess the reclassification rate and the implications of initiating lipid-lowering strategies.

## 2. Materials and Methods

We designed a pilot study as an observational, cross-sectional study. Since early 2023, SCORE-2 has been automatically calculated for all routine test requests made by primary care practitioners in the Health Department of the Hospital de San Juan [11]. Patients with low or intermediate risk were identified as candidates for Lp(a) screening and were invited to participate in the study. Once they signed the informed consent form, a blood sample was taken to measure only Lp(a) and high-sensitivity troponin I (hs-cTnI) [12]. Patients with Lp(a) > 50 mg were reclassified into a higher-risk category. Eligibility for statin treatment was assessed according to the treatment targets recommended in clinical guidelines: LDLc > 155 mg/dL for low risk, LDLc > 100 mg/dL for moderate risk, and LDLc > 70 for high risk [10,13]. The study protocol and informed consent were approved by the ethics committee of the hospital. The costs related to Lp(a) and hs-cTnI determinations were covered by the investigators’ research funds with no external funding.

The inclusion criteria were age >40 and <69 years, low or intermediate risk estimated by SCORE-2, and signing the informed consent form. Exclusion criteria were active neoplastic disease, a life expectancy of <1 year due to previous pathologies, and pregnancy.

Blood samples were obtained from a brachial vein puncture, frozen at −80 °C, and processed together. Lp(a) concentrations were quantified by particle-enhanced immunoturbidimetry on an Alinity c analyzer (Abbott Diagnostics, Abbott Park, IL 60064-3500, USA) according to the manufacturer’s instructions. The results are reported in mg/dL. Elevated Lp(a) was defined as >50 mg/dL, which is the threshold used to reclassify individuals as high risk. Determination of hs-cTnI was quantified by a chemiluminescent microparticle immunoassay (CMIA) on an Alinity i-analyzer (Abbott Diagnostics, Abbott Park, IL 60064-3500, USA) following the manufacturer’s instructions. The results are reported in ng/L [14].

We performed a sample size estimation to determine the number of patients required for the study to have 80% power to detect differences in the contrast of the null hypothesis (H_0_: μ_1_ = μ_2_) using a two-tailed Student’s *t*-test for proportions. Taking into account a significance level of 5%, a high prevalence of Lp(a) of 8%, and a 10% loss to follow-up, we estimated that the study would require at least 137 patients. These calculations were performed using G*Power 3.1. Quantitative variables are presented as median and interquartile range (IQR); differences among the four groups were assessed by the non-parametric Kruskal–Wallis test. Qualitative variables are presented as percentages, and differences were analyzed by Chi^2^. Glomerular filtration rates were calculated using the CKD-EPI formula [14]. Reclassification was represented by Sankey graphs. Statistical difference was accepted at *p* < 0.05. All analyses were performed using STATA 14.3 (StataCorp, 2009. Stata Statistical Software: Release 14. College Station, TX, USA: StataCorp LP).

## 3. Results

**Figure 1:**
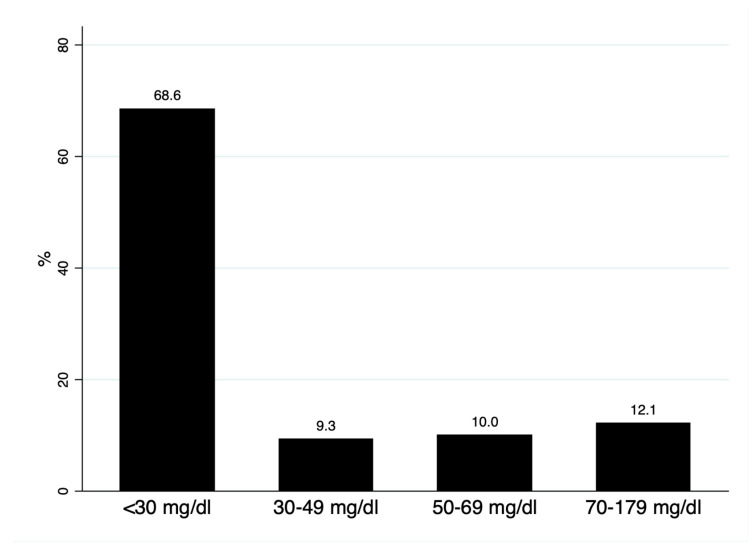
Distribution of lipoprotein(a) concentrations.

We analyzed 140 subjects, with a mean age of 54.3 (8.1) years and 62.9% women. The median Lp(a) was 15.2 (interquartile range: 6.7–44.5) mg/dL; 68.6% had Lp(a) < 30 mg/dL, 9.3% presented with levels of 30–49 mg/d, and 22.1% of the cohort had Lp(a) > 50 mg/dL (Figure 1). As shown in Table 1, no differences were observed in mean age, sex, or lipid profile in subjects with Lp(a) below or above 50 mg/dL; alkaline phosphatase (ALP) was significantly higher in subjects with Lp(a) > 50 mg/dL.

**Figure 2:**
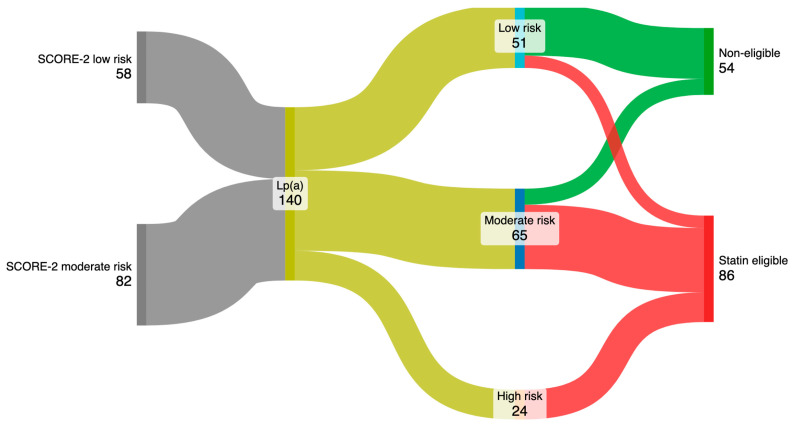
Reclassification of cardiovascular risk representation and identification of patients eligible for statins.

After incorporating Lp(a) values into the SCORE-2 assessment, 22.6% of individuals initially of low risk were reclassified to moderate risk, and 77.4% were reclassified from moderate to high risk (Figure 2). Furthermore, 19.6% of patients reclassified as low risk had LDLc > 150 mg/dL, 80% of those reclassified to moderate risk had LDLc > 100 mg/dL, and all subjects reclassified to the high-risk group had LDL > 70 mg/dL. As a consequence, 61.4% (86 subjects) were considered eligible for treatment with statins.

## 4. Discussion

The results of our community-level study show that one-fifth of subjects categorized as low or moderate risk by SCORE2 were reclassified as having a higher cardiovascular risk, and that 61.4% were eligible for statins following the Lp(a) results. These results could significantly impact the management of cardiovascular risk factors. Since our clinical features are similar to previous reports [6,7,15,16,17], we believe that these results might be representative and clinically applicable.

There is a broad consensus that Lp(a) should be determined at least once in a lifetime, since a single determination has high predictive value for the incidence of cardiovascular disease [3,9]. Lp(a) levels < 30 mg/l might be considered as non-elevated, 30–50 mg/dL as slightly elevated, and >50 mg/dL as elevated [2]; nonetheless, the inclusion criterion for some of the clinical trials was Lp(a) > 70 mg/dL [18]. Despite the evidence supporting the role of Lp(a) in cardiovascular risk, it is not widely measured due to the lack of availability or the skepticism regarding what to do with the results. We designed a pilot study for the screening of Lp(a) in our healthcare area that revealed the high prevalence of non-normal Lp(a) values, the elevated reclassification rate, and the percentage of subjects that would be eligible for statins. Similarly, once a subject has elevated Lp(a), it is recommended to test in first-line relatives [2,4]. A recent study performed in Spain showed that 60% of the relatives of patients with premature myocardial infarction and elevated Lp(a) had levels > 50 mg/dL [15]. Determining Lp(a) also has clinical implications for patients with established cardiovascular disease; while it may not change lipid treatment targets, it is associated with lower LDLc control [16] and a higher risk of recurrent ischemic events [17] and could guide prolonged dual antiplatelet therapy [19]. There are currently no therapies available to reduce Lp(a) levels below 50 mg/dL, although PCSK9-directed therapies decrease Lp(a) by 20–30%, and they could be another mechanism for reductions in major cardiovascular events observed in clinical trials [20,21,22].

One of the most direct implications of our results is that more than half of the subjects would be eligible for statins. This result underscores the potential benefits of incorporating Lp(a) screening into routine cardiovascular risk evaluation, enabling the identification of patients who may require lipid-lowering strategies even in the absence of other major risk factors. Our results are in concordance with an analysis of 10,000 subjects aged 40–69 years from the UK Biobank that found that 18% of patients have Lp(a) > 50 mg/dL (>105 nmol/L). That study achieved an estimation that revealed that the risk reclassification could induce the initiation of statin and blood pressure-lowering therapies, which would reduce the cost and burden and cardiovascular disease in this population [23]. Given the pro-atherogenic and pro-thrombotic properties of Lp(a) [24], its coexistence with elevated LDLc may accelerate vascular injury, further strengthening the rationale for early and sustained LDL-lowering interventions [10]. We conducted this pilot study prior to including Lp(a) as a routine test, which helped us to establish a protocol to detect subjects with elevated Lp(a), as is achieved with other results from the laboratory [25]. Lp(a) is mostly determined genetically and has no clear associations with lifestyle habits or other clinical conditions. Subjects with Lp(a) > 50 mg/dL in our cohort, in concordance with previous evidence [8,15,16], have similar clinical characteristics to the other subjects, which increases the relevance of an active screening plan. Subjects with Lp(a) > 50 mg/dL had a higher prevalence of moderate cardiovascular risk, and, therefore, the screening might be even more effective in this group.

Our study has some limitations. Firstly, it was a cross-sectional and single-center study that can only describe associations but not causality. Secondly, subjects were invited to participate, which induced a selection bias. Thirdly, although Lp(a) was measured using a standardized immunoturbidimetric assay, the results were expressed in mg/dL; molar units (nmol/L) are increasingly recommended to improve comparability across studies and to align with emerging therapeutic trial criteria. Fourthly, sample size was relatively modest and the participants were from a specific geographical region; this may not reflect the distribution of Lp(a) levels or reclassification rates in other populations with different ethnic, genetic, or lifestyle backgrounds; similarly, we could not adjust for certain potential confounding factors, specific dietary habits, family histories of premature cardiovascular disease, or concomitant inflammatory conditions, which may influence cardiovascular risk independently of Lp(a). Finally, there was no longitudinal follow-up available to assess whether reclassification based on Lp(a) translated into improved clinical outcomes, such as reductions in cardiovascular events, following changes in lipid-lowering therapy; this is planned for the coming years. Since clinical features are similar to previous reports [6,7,8,15,16], we believe that our results might be representative and clinically meaningful.

## 5. Conclusions

Our results show that, as a result of Lp(a) testing, 22.1% of subjects classified as having low or moderate cardiovascular risk were reclassified into a higher-risk category, and 62.4% were considered eligible for statin treatment. The results underscore the potential benefits of incorporating Lp(a) screening into routine cardiovascular risk evaluation, enabling the identification of patients who may require lipid-lowering strategies even in the absence of other major risk factors.

## Figures and Tables

**Table 1:** Clinical characteristics of the population according to Lipoprotein(a) [Lp(a)] levels.

	Total	Lp(a)		
		<50 mg/dL	>50 mg/dL	*p*
N	140	109 (77.9%)	31 (22.1%)	
Lp(a) (mg/dL)	15.2 (6.7–44.5)	10.3 (5.4–19.9)	72 (55.6–89.6)	<0.01
Low risk (SCORE2)	58 (41.4%)	51 (46.8%)	7 (22.6)	0.02
Moderate risk (SCORE2)	82 (58.6%)	58 (53.2%)	24 (77.4%)	0.02
Age	54.3 (8.1)	54.3 (8.3)	53.7 (7.6)	0.72
Women	88 (62.9%)	66 (60.6%)	22 (71.0%)	0.29
Current smoking	17.1%	20.2%	6.5%	0.07
Systolic BP (mmHg)				0.27
110–119 mmHg	19.3%	22.6%	20.0%	
120–139 mmHg	75.2%	67.7%	73.5%	
140–159 mmHg	5.7%	5.5%	6.5%	
160–179 mmHg	0.0%	3.2%	0.7%	
Leucocytes (10^9^/L)	6.3 (1.6)	6.4 (1.6)	6.1 (1.6)	0.33
Hemoglobin (g/dL)	14.1 (1.3)	14.1 (1.3)	14.2 (1.2)	0.67
Platelet count (U/L)	234,000 (202,000–261,000)	234,000 (201,500–266,000)	224,000 (210,000–251,000)	0.82
Glucose (mg/mL)	88.5 (14.1)	88.2 (15.4)	89.6 (8.4)	0.70
Creatinine (mg/dL)	0.82 (0.2)	0.82 (0.2)	0.81 (0.2)	0.67
GFR (ml/min/1.72 m^2^)	89.3 (14.1)	88.9 (14.4)	90.7 (13.3)	0.62
Total cholesterol (mg/dL)	203.2 (34.7)	202.5 (35.8)	205.6 (31.3)	0.67
LDLc (mg/dL)	124.5 (30.2)	124.0 (31.1)	126.0 (27.1)	0.75
HDLc (mg/dL)	59.0 (15.1)	58.3 (15.4)	61.5 (14.0)	0.30
Triglycerides (mg/dL)	85 (60–105)	85 (60–125)	85 (65–95)	0.50
Non-HDL cholesterol (mg/dL)	144.8 (33.78)	145.0 (35.5)	145.1 (27.2)	0.90
Hs-Troponin (pg/mL)	1.5 (1.5–2.4)	1.5 (1.5–2.4)	1.5 (1.5–2.6)	0.60
ALP (U/L)	85.8 (32.5)	81.5 (30.7)	99.4 (34.8)	0.01
HbA1c	5.6 (0.3)	5.5 (0.3)	5.6 (0.4)	0.33

## Data Availability

The datasets presented in this article are not readily available because they were collected by the investigators. Requests to access the datasets should be directed to acorderofort@gmail.com.

## Abbreviations

The following abbreviations are used in this manuscript:

hs-cTnI	High-sensitivity troponin
LDLc	Low-density lipoprotein cholesterol
Lp(a)	Lipoprotein(a)
